# “Barsati,” or Atrophic Carcinoma

**Published:** 1887-01

**Authors:** Richard W. Burke

**Affiliations:** Cawnpore, India


### Art. VIII.—"BARSATI," OR ATROPHIC CARCINOMA.

BY RICHARD W. BURKE, V. S., A. D. V.
*Cawnpore, India.*

(Continued from page 376, Vol. VII.)

Many observers agree in stating that Barsati sores are very liable to be immediately developed in the seats of abrasions, and that small sores take on in India a fungous or ulcerative character like Barsati sores. Mr. Hart speaks of this as occurring commonly during the rains in connection with the wounds and galls of the horse. Armstrong notices the same thing. And we have only recently recorded several similar instances—which would not show a parasitic origin in respect of this disease. There can be little question that disorder of the general nutrition induced by climate is one element in the production of Barsati and its allies. The parts attacked are those most exposed to injuries or irritation: *example*, angles of the mouth, the lachrymal region, the prepuce, inner aspect of the fetlocks, &c. And we are not without analogical evidence of similar disease being induced in like manner in other species, and in some other countries. French medical literature abounds in instances of such tissue changes being the results of climatic influences. In the West Indies, in China, and in some other countries, it has been observed that sores take on not a suppurative or ulcerative action, though they do this sometime, but frequently are succeeded by an hypertrophous growth of the fibrous tissue as the result of such influences. It is clearly shown by these results, therefore, that the causes which have been supposed to engender them are not such as can, in any intelligible way, favor the introduction of germs from without the body. Thus, in this disease, the continued irritation of tears over the lachrymal surface, or of secretions within the prepuce, or of the bit against the angles of the mouth of the horse, has been observed to be followed by the occurrence of Barsati in these parts in such a number of cases as to justify the inference that it has been the starting point of the disease. Such growths arise, then, from a peculiar perversion of the normal nutritive process, allied to those modifications which we have been induced to suppose are the causes of the more peculiar kinds of common growths; but in the case of malignant growths, the perversion is much greater in degree, and shows itself not only in its origin, but in its whole subsequent history. The irritations connected with the bit at the angles of the mouth, with the secretions in the prepuce, and the habit of "brushing" at the fetlocks, &c., may induce this extraordinary alteration in the molecular nutrition of these parts, leading to the disease under consideration. We have had several times the opportunity of examining portions of skin affected with Barsati in the earliest stages, and the anatomical changes present in them have been uniform and simple. There is an area of increased vascularity, over which a dense infiltration with small cells is seen, placed just beneath the epithelium. The latter is thickened and piled up in disorderly fashion. The cells of the rete are undergoing rapid multiplication, the lower layers being crowded, and in places heaped up in masses or little *balls* which, often attaining to a large size readily visible to the naked eye, undergo calcareous deposition, and acquire a hardness which has likened them to the stone known in India under the name *kunkur*. Thus the whole process of normal formation of the skin is at a disturbance. Whether the excitant which causes the disturbance is engendered by chronic irritation of the part, or whether it is only rendered locally active by it under certain constitutional * conditions are questions which remain at present unsolved in pathology. But the degree of evolution to which a new formation may attain, must plainly depend, to a large extent upon the nature of the excitement. The more it resembles the normal stimulus to growth and development, the more perfect and limited will be the development of the new growth; and the more intense the excitant, the more embryonic and unlimited the growth.

Those who are contending for a proof of the parasitic theory

* The significance of desquamation following the administration or use of certain drugs, such as turpentine, perhaps capaiba, &c., may have a good deal to teach yet about the law under which cell changes take place locally, as a result of constitutional causes.

of this disease, seem to be driven to the choice of one alternative—Is it, or is it not communicable? Mr. Smith alone has succeeded,* after experiencing many failures,† in inoculating Barsati from one animal the subject of it to another that was not. And if we review the general experience in regard to experiments in this direction concerning both cancer of man and Barsati as it occurs in the horse, we find that the results of such experience differ in respect of neither. An experiment of Professor Langenbeck was supposed to have proved that the cancerous pulp containing the cancer cells is capable of propagating cancer in man and animals on being injected into the veins, but the attempt has been frequently made by others without any result. So in regard to Barsati; excepting the above instance, all other experiments by other Veterinary Surgeons have uniformly failed to produce the disease, although enough experiments have now been made in this direction.

The fact that the disease can be induced by inoculation, moreover, does not prove that the cause of the disease is derived from without the body, since the very cells of the body themselves, as changed in disease, can be transplanted from body to body and induce furunculoid and ulcerative mischief; in other words, there is nothing in the aspect of the disease itself, nor in the facts of the inoculated disease, to show that its cause is very likely to be parasitic. A direct transmission of Barsati has been proved impossible; and it by no means follows that one or two exceptional successes which are recorded were entirely due to the inoculations, and were not developed in the ordinary course of things, seeing that simple wounds in India so often terminate in Barsati. I do not wish to say anything in discouragement of experiments, seeing that I have probably tried inoculations on as many animals myself; but individual results should include general results. It is also probable that constitutional predisposition, as from previous attacks of disease, when present, may favor the success of an inoculation. We must at present explain differently the connection between such successes and the causation of the disease under consideration. We, at any rate, know that the

*Veterinary Journal, 1884.

†Ibid, 1879, 1881.

accumulated experience of the veterinarians of thirty odd years shows that experiment in this direction is by no means isolated but is one of a series, many of which have been published in the pages of our professional journals. We may study Mr. Smith's success from two points of view, as showing the influence of constitutional tendency upon local disease processes, and the influence of local irritation upon general constitutional states. It is known that in a very large number of cases where simple wounds been seen to be followed by Barsati sores, the, constitution of the animal was at fault, as shown by the history of many cases. Although the actually demonstrable lesions of this disease appear to travel from local to systemic centers, there is yet every reasonable probability that constitutional tendency, especially if favored by climatic causes, may be the starting point of genuine Barsati. The local manifestations may be regarded as expressions of a general dyscrasia; and an animal which from an injury or irritation gets indubitable Barsati may be held to be predisposed from causes latent in the system, which may appear healthy or even produce such results on the body as may be designated for the time "robust."

Thus far we have justified the contention, I think, that the disease originates by means of primary changes in the skin of necrotic character; and although the "fungus" theory has long been in high favor with some writers, there is an abundance of valuable and fruitful work to be done before these questions can be definitely settled, the disease having, at one time or another, been mistaken for most, if not all, of the chronic ulcers and sores of India, including Delhi-Boil, Keloid, Rodent Ulcer and Ichthyosis of the horse. We have no idea how common it is, at first sight, to mistake any of the above mentioned diseases for Barsati, and how necessary it is, in dealing with any disease, to recognize and watch the clinical differences that are presented. The secondary changes in internal organs we have recorded in many cases of long-standing Barsati are due to proliferation and malnutrition of the parts concerned, and necrotic changes locally taking place, owing to the discharge of necrotic tissue into the circulation. This *metastasis* never manifests itself early in the course of the disease, but shows itself only after a long interval, as proved by numerous autopsies we have now made.

Many observers exalt special remedies in the treatment of Barsati, and, as usual, each produces a list of cures in favor of his favorite application. Further evidence of the difficulty of settling this point is afforded by general experience, which disproves the utility of these cures, and shows that their benefits have been over-estimated. The results of treatment, therefore, still further assimilate it with the cancer of man.

Not a few cases of Barsati become, after a shorter or longer time, arrested and retrogressive, and, ultimately, to all appearances, cured. These apparent cures are not always permanent, but after intervals of six months, a year, or more, a similar affection is started again, either in the same or in another part, or in both, when it contaminates the internal organs, or becomes again *atrophic* and is arrested, another striking resemblance between it and the form of human cancer known on the continent of Europe under the name *Atrophic Carcinoma.* Some pathologists call this *Recurrent* Fibroid,* a vague term applied to many different species. For a tumor to be called fibroid it is not enough that it should contain fibrous tissue; it must also contain no other tissue. A fibrous matrix is common to nearly all tumors; and Barsati has a fibrous stroma which predominates in many parts of its structure, but it always contains in its mesh-work cells; whereas true fibroids are composed solely of fibrous tissue. Moreover, although the distinctions of the various forms of cancer are useful and wise, and practical, and founded on natural laws, still there are cases in which these different forms pass into each other, and in which all these peculiarities may be seen affecting the same patient. "These cases of combination" says a recent writer on cancer, "are sufficiently rare to make the distinctions valuable in practice, but they at the same time show the common character of all these forms of disease." Various other names have been given to this form of cancer, as for example, *contracting cancer, cicatrical cancer, &c.*, each of which indicates some prominent feature of the disease. The cells, in this form of the disease, appear to be very short-lived, for they are scarcely formed before they commence to decay

* The term "recurrent" here is misleading, inasmuch as all malignant growths are recurrent.

(*Billroth*); but peripherally the slight cell-infiltration constantly extends; hence complete disappearance of the disease very rarely, if ever, takes place. Some surgeons even object to the term cancer being applied to epithelioma, but this objection is not valid, being founded on mere physical differences of no importance.

Mr. Hart writes—"After an *indefinite* period of immunity the disease (Barsati) reappears." We know the history of several cases in which the disease recurred after a cessation of three years, and in one case, recently treated, after a period of four years, whilst Hutchinson, Professors Billroth, Buchanan, and Dr. Whitehead give instances of immunity after operation and other modes of treatment for cancer, extending to three, five, nine, and fifteen years in the case of man.* The tendency to fibroid development in excess of cell formation is one of the most potent agencies in giving respite, and, in some cases, towards arrest and apparent care of the disease.

The original starting-point of cancer is a question on which opinions are extremely various. Virchow says, the disease commences in the connective tissue of the skin: Thiersch contends that it originates in the sebaceous glands in conjunction with projections of the rete into the cutis; while Koster argues that the disease begins in neither of these ways, but by a change in the endothelium of the vessels alone and without participation of the connective tissue. Koster's view is favored by Billroth, who thinks that the principal seat of the activity of cancer is the endothelium of the lymphatics of the skin. Rindfleisch inclines to the view of Thiersch. Thus we see that the precise origin of cancer, so far as is at present determined, amounts to conflicting theories. If any other tissue were present, no doubt some one would advance it as the origin of the disease. There is little doubt but that all the tissues are more or less affected, and in our diagnosis of this disease we must not go by the changes presented by any one particular structure of the skin, but by the changes presented by all the structures taken collectively, and these considered in reference to the clinical aspects of the disease.

The above extract of opinions shows the obscurity of the

*(Lancet, October 29, 1881).

question and the consequent general uncertainty and conflicting opinions that prevail respecting the origin of cancer, and offer, perhaps, the best apology I can allege for intruding my opinions and conclusions as to the actual causes of Barsati in the horse.

(To be Continued.)

